# The impact of COVID-19 on a South African pediatric cardiac service: implications and insights into service capacity

**DOI:** 10.3389/fpubh.2023.1177365

**Published:** 2023-05-10

**Authors:** Thomas Aldersley, Andre Brooks, Paul Human, John Lawrenson, George Comitis, Rik De Decker, Barend Fourie, Rodgers Manganyi, Harold Pribut, Shamiel Salie, Lenise Swanson, Liesl Zühlke

**Affiliations:** ^1^Department of Pediatrics and Child Health, Pediatric Cardiology, University of Cape Town, Cape Town, South Africa; ^2^Red Cross War Memorial Children’s Hospital, Cape Town, South Africa; ^3^Chris Barnard Division of Cardiothoracic Surgery, University of Cape Town, Cape Town, South Africa; ^4^Groote Schuur Hospital, Cape Town, South Africa; ^5^Division of Pediatric Cardiology, Department of Pediatrics and Child Health, University of Stellenbosch, Cape Town, South Africa; ^6^Tygerberg Hospital, Cape Town, South Africa; ^7^Department of Pediatrics and Child Health, Pediatric Intensive and Critical Care Unit, University of Cape Town, Cape Town, South Africa; ^8^South African Medical Research Council, Cape Town, South Africa

**Keywords:** COVID-19, pediatric cardiology, cardiothoracic surgery, cardiac surgery, congenital heart disease, service capacity

## Abstract

**Background and Objectives:**

The Western Cape public pediatric cardiac service is under-resourced. COVID-19 regulations are likely to have long-term effects on patient care but may provide insight into service capacity requirements. As such, we aimed to quantify the impact of COVID-19 regulations on this service.

**Methods:**

An uncontrolled retrospective pre-post study of all presenting patients over two, one-year periods; the pre-COVID-19 period (01/03/2019–29/02/2020) and the peri-COVID-19 period (01/03/2020–28/02/2021).

**Results:**

Admissions decreased by 39% (624 to 378) and cardiac surgeries decreased by 29% (293 to 208) in the peri-COVID-19 period, with an increase in urgent cases (PR:5.99, 95%CI:3.58–10.02, *p* < 0.001). Age at surgery was lower in the peri-COVID-19 period, 7.2 (2.4–20.4) vs. 10.8 (4.8–49.2) months (*p* < 0.05), likewise, age at surgery for transposition of the great arteries (TGA) was lower peri-COVID-19, 15 (IQR:11.2–25.5) vs. 46 (IQR:11–62.5) days (*p* < 0.05). Length of stay 6 (IQR:2–14) vs. 3 days (IQR:1–9) (*p* < 0.001), complications (PR:1.21, 95%CI:1.01–1.43, *p* < 0.05), and age-adjusted delayed-sternal-closure rates (PR:3.20, 95%CI:1.09–9.33, *p* < 0.05) increased peri-COVID-19.

**Conclusion:**

Cardiac procedures were significantly reduced in the peri-COVID-19 period which will have implications on an overburdened service and ultimately, patient outcomes. COVID-19 restrictions on elective procedures freed capacity for urgent cases, demonstrated by the absolute increase in urgent cases and significant decrease in age at TGA-surgery. This facilitated intervention at the point of physiological need, albeit at the expense of elective procedures, and also revealed insights into capacity requirements of the Western Cape. These data emphasize the need for an informed strategy to increase capacity and reduce backlog whilst ensuring minimal morbidity and mortality.

**Graphical Abstract:**
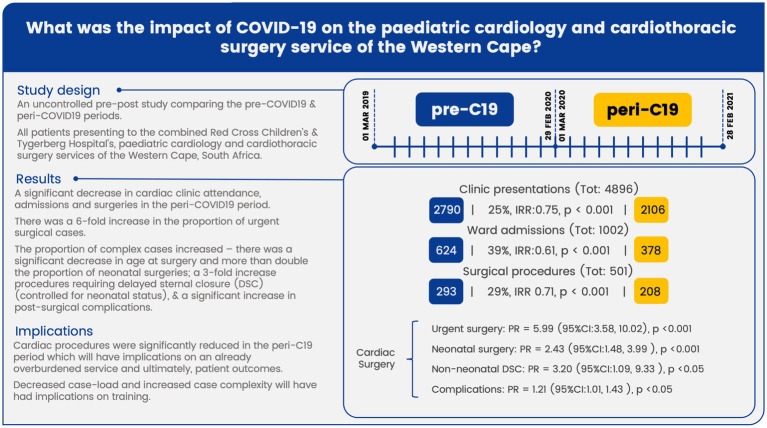

## 1. Introduction

The South African pediatric cardiology and cardiothoracic surgery service is in general under-resourced. Although better resourced than most South African provinces, the Western Cape service, by international standards, is still considered to be under-resourced and any interruption to this service may have long term effects. We aimed to quantify the impact of COVID-19 regulations on the Pediatric Cardiology service of the Western Cape and the pediatric division of the Christian Barnard Division of Cardiothoracic Surgery.

### 1.1. Background

In response to the growing pandemic and the identification of COVID-19 cases in South Africa, on the 18 March 2020, the South African government restricted domestic and international travel, gatherings of more than 50 people and closed schools (1–3). This was followed by a 21-day level-5 lockdown on the 27 March 2020. Level-5 lockdown entailed restrictions on the movement of persons and goods, the prohibition of public transport, and the closure to the public of places and premises not essential to the provision of essential services or goods (4). Level 5 lockdown was extended for a further 14 days before the implementation of a staged de-escalation to lockdown level 1 almost 1 year later, on the 1st of March 2021. Although the health care sector was exempt from level 5 regulations (4, 5), most provincial health departments and private hospital groups elected to suspend the provision of elective and non-urgent interventions and surgery (6, 7).

The Red Cross War Memorial Children’s hospital and Tygerberg Hospital serve as the combined pediatric cardiology and cardiothoracic surgery referral center for the population of the Western Cape, parts of the Eastern and Northern Cape and, serves a population of approximately nine million people (8). Annually this service typically performs around 120 interventional cardiac catheterisations, 300 cardiac surgeries and has a surgical waiting list of more than 200 patients (8). Although essential, many of these procedures are performed electively and as such would have been curtailed by the regulations described above.

Local and international studies show that COVID-19 restrictions have negatively impacted health services and lead to significant reductions in in-patient procedures (6, 9). In the Western Cape province of South Africa, general surgical procedures decreased by 44% in the peri-COVID-19 period (10). Internationally, an Italian multi-center study showed a 53% reduction in adult cardiac surgery for the 2020 peri-COVID-19 period when compared with the 2019 pre-COVID-19 period (11). Similarly, at the Seattle Children’s Hospital there was a 26% reduction in pediatric cardiac surgery and a 44% reduction in non-surgical pediatric cardiac interventions during the peri-COVID-19 period (12).

The South African public pediatric cardiology and cardiothoracic surgery service is in general considered to be under-resourced with insufficient nurses, allied staff, pediatric cardiologists and cardiothoracic surgeons to meet the needs of the population (8, 13–15). Although better resourced than most South African provinces, the Western Cape service, by international standards (16), is still considered to be under-resourced, with less than half the recommended 18 pediatric cardiologists for the population of 9 million and insufficient cardiothoracic surgeons to meet the needs of the estimated >550 Western Cape patients requiring open heart surgery annually (this number excludes patients from the Eastern and Northern Cape seen in the Western Cape service) (15–18). As such, any COVID-19-related backlog cannot be cleared by returning to pre-COVID-19 capacity (19). Additional capacity must be leveraged and evidence based strategies implemented to minimize complications and deaths in patients awaiting treatment. In addition, the COVID-19-related restrictions to the provision of non-urgent cardiac procedures, may reveal insights into Western Cape public pediatric cardiac service capacity. As such, this paper aims to quantify the impact of COVID-19 regulations on the public pediatric cardiology and cardiothoracic surgery service of the Western Cape, and to inform future studies and interventions to address the resultant backlog.

## 2. Materials and methods

We conducted an uncontrolled pre-post study of pediatric cardiology and cardiothoracic surgery patients presenting to the Department of Health, Pediatric Cardiology service of the Western Cape and the Chris Barnard Division of Cardiothoracic Surgery over two, one-year periods. The pre-COVID-19 period (01/03/2019–29/02/2020) and the peri-COVID-19 period (01/03/2020−28/02/2021).

Data were collected retrospectively from: the Chris Barnard Division of Cardiothoracic Surgery, pediatric cardiac surgery patient management and auditing system, the Pediatric Cardiology service of the Western Cape Red Cross Children’s Hospital cardiology patient database and associated preoperative waiting list, the International Quality Improvement Collaborative for Congenital Heart Disease (IQIC) Red Cross Children’s Hospital database, the Red Cross Children’s Hospital Pediatric Intensive and Critical Care Unit Clinical Database and the Western Cape Department of Health Clinicom^™^ administration system. Institutional review board approval for this study was obtained from the University of Cape Town, Human Research Ethics Committee (HREC). A waiver of informed consent was granted as the study is a *post hoc* analysis of existing HREC approved registries and databases.

The primary objectives were to quantify changes in the number of cardiac out-patient department (OPD) visits, ward admissions, surgical procedures and cardiac catheterisations. Additionally, we compared changes in age, the distribution of diagnoses, OPD appointment attendance rates, ward admission duration and level of care, surgical procedure type, urgency of surgical care, surgical risk or complexity, using the Risk Adjustment for Congenital Heart Surgery 1(RACHS-1) score (20), surgical complication and mortality rates, and the number of cardiac catheterisation interventions and complications.

Poisson regression models were fitted to determine the incidence rates and incidence rate ratios (IRRs) for the dichotomized data: total OPD presentation, total ward admissions, total surgical procedures, and total cardiac catheterisations, modelled independently as the response variable, and the explanatory variable constituting the date, which was factorized into two levels, the pre-COVID-19 period or the peri-COVID-19 period, described above. Wilcoxon rank sum tests were used to compare differences in median age between periods. For categorical data, prevalence ratios between periods were calculated for each category individually, 95% confidence intervals were calculated, and *p*-values were generated using Chi-square and Fisher’s exact tests where appropriate. Statistical analysis was carried out in R (version 4.2.0, R Foundation) (21).

## 3. Results

**Figure 1:**
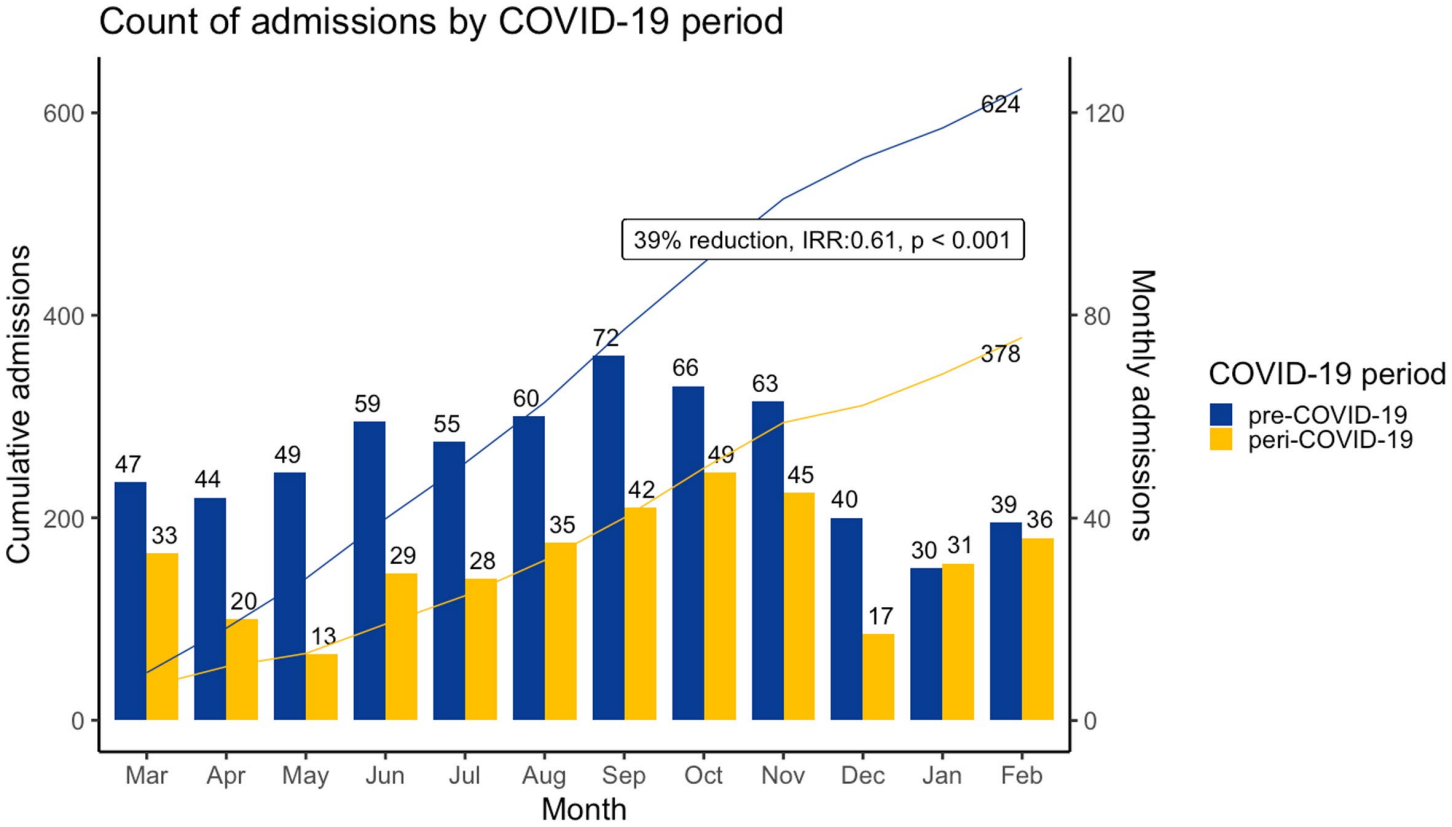
Count of cardiac admissions by COVID-19 period, cardiac admissions were significantly lower (IRR: 0.61, 95%CI: 0.48–0.62 *p* < 0.001) in the peri-COVID-19 period.

Combined surgical and medical cardiac admissions (Table 1; Figure 1) decreased by 39% from 624 in the pre-COVID-19 period to 378 in the peri-COVID-19 period. Median age at admission was similar between periods, 2.7 years (Interquartile Range [IQR]: 0.8–6.8) pre-COVID-19 and 1.81 years (IQR: 0.5–6.2) peri-COVID-19. With a significant decrease in patients aged 5 to 12 years (PR: 0.78, 95%CI: 0.63–0.98, *p* < 0.05) and a significant increase in children aged 12 to 19 years (PR: 1.85, 95%CI: 1.09–3.12, *p* < 0.05). The distribution of diagnoses was similar for both periods; structural CHD predominated, with 535 (64.8%) cases pre-COVID-19 and 328 (67.5%) cases peri-COVID-19, followed by myocardial or pericardial disease, with 71 (8.6%) cases pre-COVID-19 and 30 (6.2%) cases peri-COVID-19. Admission-duration was significantly longer (*p* < 0.001), 3 days (IQR: 1–9) in the pre-COVID-19 period versus 6 (IQR: 2–14) peri-COVID-19. ICU admissions decreased from 239 to 167, with a non-significant relative increase in ICU admissions (PR: 1.14; 95% CI: 0.98–1.33, *p* = 0.085) during the peri-COVID-19 period. Length of ICU admission was similar for both periods; 3 days (IQR: 2–6.5) for the pre-COVID-19 period and 4 days (IQR: 2–7.5) for the peri-COVID-19 period.

**Table 1:** Admission and OPD presentation data.

Period	Admissions					OPD presentations				
	Pre-COVID-19		Peri-COVID-19		% Reduction, *p* value	Pre-COVID-19		Peri-COVID-19		% Reduction, *p* value
**Total**	624		378		39%, *p* < 0.001	2,790		2,106		25%, *p* < 0.001
**Age**	**Median**	**IQR**	**Median**	**IQR**	***p* value**	**Median**	**IQR**	**Median**	**IQR**	***p* value**
Age, years (median [IQR])	2.7	(0.8–6.8)	1.8	(0.5–6.2)	*p* = 0.19	4.4	(1.3–8.6)	4.1	(1–8.75)	*p* < 0.05
Age, categorical	**Count**	**(%)**	**Count**	**(%)**	**RR &95% CI, *p* value**	**Count**	**(%)**	**Count**	**(%)**	**RR &95% CI, *p* value**
Neonate (0,27d]	28	5	26	7	1.53 (0.91, 2.57), *p* = 0.10	4	0.1	4	0.2	1.32 (0.33, 5.29), *p* = 0.97
Infant [28d,12 m)	179	29	121	32	1.12 (0.92, 1.35), *p* = 0.27	578	21	510	24	1.17 (1.05, 1.30), *p* < 0.05
Toddler [1,2y)	77	12	49	13	1.05 (0.75, 1.47), *p* = 0.77	324	12	236	11	0.96 (0.82, 1.13), *p* = 0.66
Early childhood [2,5y)	132	21	67	18	0.84 (0.64, 1.09), *p* = 0.19	586	21	419	20	0.95 (0.85, 1.06), *p* = 0.34
Middle childhood [5,12y)	183	29	87	23	0.78 (0.63, 0.98), *p* < 0.05	970	35	694	33	0.95 (0.88, 1.03), *p* = 0.19
Early adolescence [12,19y)	25	4	28	7	1.85 (1.09, 3.12), *p* < 0.05	322	12	242	11	1.00 (0.85, 1.16), *p* = 0.96
Late adolescence [19,21y)	1	0	0	0	na	2	0.1	0	0	na
**Diagnosis**	**Count**	**(%)**	**Count**	**(%)**	**RR &95%CI, *p* value**	**Count**	**(%)**	**Count**	**(%)**	**RR &95%CI, *p* value**
Structural CHD	535	65	328	68	1.04 (0.96, 1.13), *p* = 0.33	2,359	67	1776	65	0.97 (0.93, 1.00), *p* = 0.08
Myocardial & pericardial disease	71	8.6	30	6.2	0.72 (0.48, 1.08), *p* = 0.11	276	7.8	131	4.8	0.61 (0.50, 0.75), *p* < 0.001
Acquired heart disease	35	4.2	20	4.1	0.97 (0.57, 1.66), *p* = 0.91	103	2.9	88	3.2	1.10 (0.83, 1.45), *p* = 0.51
Associated syndromes	21	2.5	10	2.1	0.81 (0.38, 1.70), *p* = 0.57	87	2.5	86	3.1	1.27 (0.95, 1.70), *p* = 0.11
Cardiac arrhythmias	16	1.9	4	0.8	0.42 (0.14, 1.26), *p* = 0.11	58	1.6	42	1.5	0.93 (0.63, 1.38), *p* = 0.72
Normal heart	11	1.3	7	1.4	1.08 (0.42, 2.77), *p* = 0.87	56	1.6	53	1.9	1.22 (0.84, 1.77), *p* = 0.30
Other	136	17	87	18	1.09 (0.85, 1.39), *p* = 0.51	591	17	570	21	1.24(1.12, 1.38), *p* < 0.001
Total diagnoses^a^	825		486			3,530		2,746		
**Ward - Length of stay**	**Median**	**IQR**	**Median**	**IQR**	**95% CI, *p* value**					
Ward length of stay (d)	3	(1-9)	6	(2-14)	*p* < 0.001	–		–		–
**ICU**	**Count**	**(%)**	**Count**	**(%)**	**95% CI, *p* value**					
ICU admissions	239	38	167	44	1.15 (0.99, 1.34), *p* = 0.07	–		–		–
ICU deaths	6	2.5	7	4.2	1.67 (0.57, 4.88), *p* = 0.34	–		–		–
**ICU**	**Median**	**IQR**	**Median**	**IQR**	**95% CI, *p* value**					
ICU length of stay (d)	3	(2–6.5)	4	(2–7.5)	*p* = 0.34	–		–		–

na = not applicable.

a Multiple diagnoses permitted per patient, but only 1 of each diagnosis category. Associations highlighted in green are statistically significant.

Out-patient visits (Table 1) decreased by 25% from 2,790 pre-COVID-19 to 2,106 in the peri-COVID-19 period. Median age at presentation was significantly lower in the peri-COVID-19 period (*p* < 0.05). Median age was 4.4 years (IQR: 1.3–8.6) pre-COVID-19 and 4.1 years (IQR: 1.0–8.75) peri-COVID-19, with a significant increase in the infant age group (PR: 1.17, 95%CI: 1.05–1.30, *p* < 0.05). The proportions of diagnosis categories were similar between periods except for a significant decrease in the proportion of myocardial & pericardial disease seen as out-patients (PR: 0.61, 95% CI: 0.50–0.75, *p* < 0.001).

**Figure 2:**
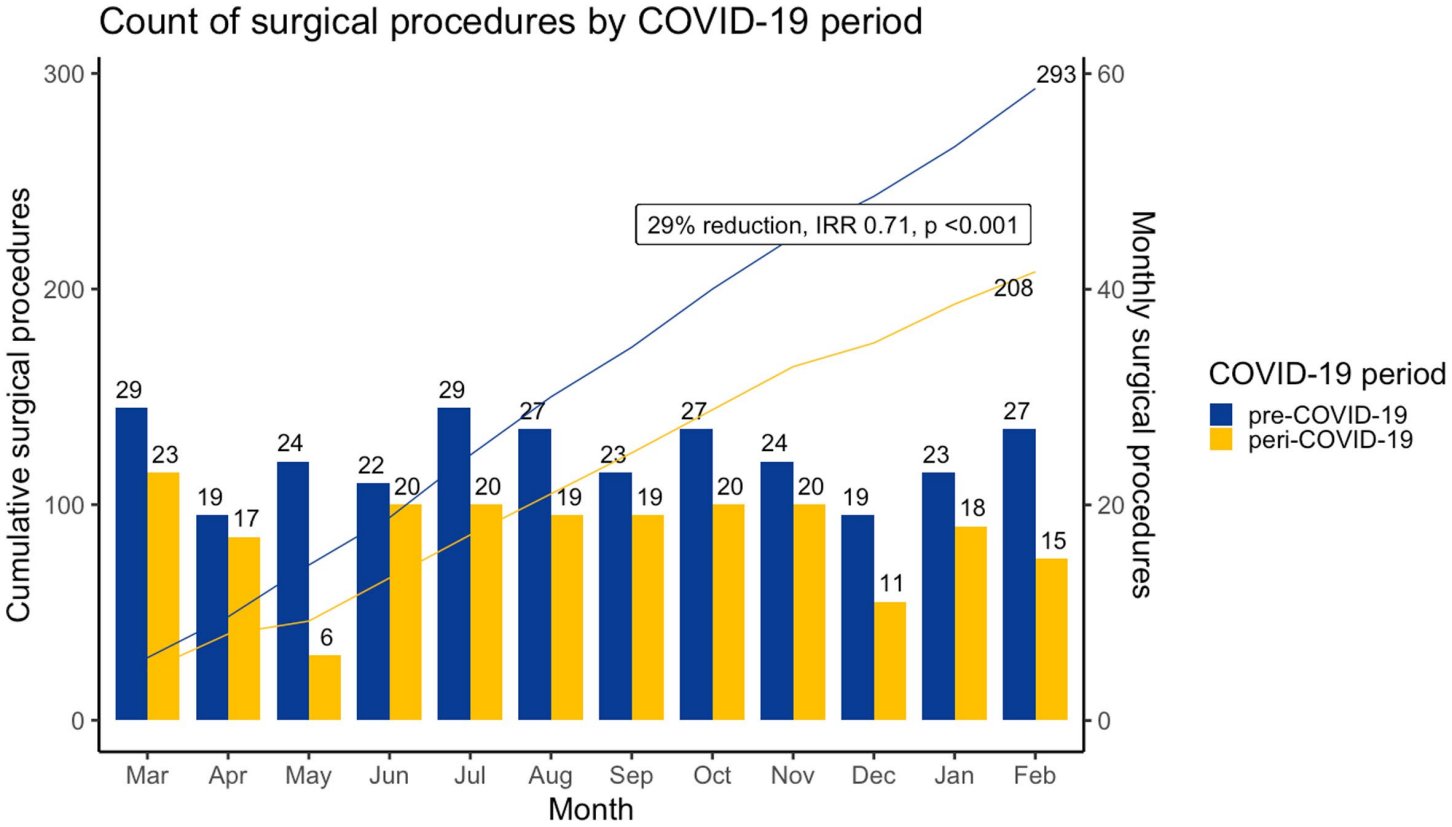
Count of cardiac surgeries by COVID-19 period, cardiac surgeries were significantly lower (IRR: 0.71, 95%CI: 0.53–0.89, *p* < 0.001) in the peri-COVID-19 period.

Cardiac surgeries decreased by 29% from 293 to 208 (Table 2, Figure 2), with significant changes in the proportion of urgent (PR: 5.99; 95% CI: 3.58–10.02, *p* < 0.001) and elective cases (PR: 0.73, 95% CI: 0.66–0.82, *p* < 0.001). Median age at surgery was significantly lower, 7.2 months (2.4–20.4) peri-COVID-19 versus 10.8 (4.8–49.2) months pre-COVID-19 (*p* = 0.001), related to a significant increase in neonatal procedures (PR: 2.43, 95% CI: 1.48–3.99, *p* < 0.001) and decrease in patients aged 5 to 12 (PR: 0.55, 95% CI: 0.34–0.88, *p* < 0.05). Procedure type was similar across periods with septal defects (pre-COVID-19:116/293, 40% vs. peri-COVID-19: 79/208, 38%), right heart lesions (pre-COVID-19: 44/293, 15% vs. peri-COVID-19: 33/208, 16%), thoracic arteries and veins (pre-COVID-19: 37/293, 13% vs. peri-COVID-19: 36/208, 17%), and left heart lesions (pre-COVID-19: 22/293, 8% vs. peri-COVID-19: 15/208, 7%) procedures predominating (Table 2). Sub-analysis of surgery for transposition of the great arteries (TGA) revealed similar rates between periods (pre-COVID-19: 19/293, 6% vs. peri-COVID-19: 22/208, 11%), however, median age at TGA surgery was significantly lower in the peri-C19 period, 15 (11.2–25.5) vs. 46 (11–65.5) days (*p* = 0.047).

**Table 2:** Surgical data.

Period	Pre-COVID-19		Peri-COVID-19		% Change
**Surgeries (total)**	293		208		29%, IRR 0.71, *p* < 0.001
**Age**	**Median**	**IQR**	**Median**	**IQR**	**RR & 95%CI, *p* value**
Age, months (median [IQR])	10.8	(4.8–49.2)	7.2	(2.04–20.4)	*p* = 0.001
Age, categorical	**Count**	**(%)**	**Count**	**(%)**	**RR & 95%CI, *p* value**
Neonate (0,27d]	22	7	38	18	2.43 (1.48, 3.99), *p* < 0.001
Infant [28d, 12 m)	138	47	94	45	0.96 (0.79, 1.16), *p* = 0.673
Toddler [1, 2)	25	8	28	13	1.58 (0.95, 2.63), *p* = 0.077
Early childhood [2, 5)	45	15	22	10	0.69 (0.43, 1.11), *p* = 0.121
Middle childhood [5, 12)	54	18	21	10	0.55 (0.34, 0.88), *p* = 0.010
Early adolescence [12, 19)	9	3	5	2	0.78 (0.27, 2.30), *p* = 0.655
**Procedure Kingdom**	**Count**	**(%)**	**Count**	**(%)**	
Septal defects	116	40	79	38	0.96 (0.77, 1.20), *p* = 0.716
Right heart lesions	44	15	33	16	1.06 (0.70, 1.60), *p* = 0.795
Thoracic arteries and veins	37	13	36	17	1.37 (0.90, 2.09), *p* = 0.143
Left heart lesions	22	8	15	7	0.96 (0.51, 1.81), *p* = 0.900
Transposition of the great arteries	19	6	22	11	1.63 (0.91, 2.94), *p* = 0.100
Palliative procedures	20	7	8	4	0.56 (0.25, 1.25), *p* = 0.100
Pacemaker procedures	15	5	5	2	0.47 (0.17, 1.27), *p* = 0.126
Pulmonary venous anomalies	10	3	5	2	0.70 (0.24, 2.03), *p* = 0.514
Single ventricle	5	2	2	1	0.56 (0.11, 2.88), *p* = 0.754
Miscellaneous procedures	3	1	1	0	0.47 (0.05, 4.48), *p* = 0.870
Conduit operations	1	0	1	0	1.41 (0.09, 22.39), *p* = 1.000
Cor Triatriatum	0	0	1	0	na
DORV	1	0	0	0	na
**Urgency**	**Count**	**(%)**	**Count**	**(%)**	**RR & 95%CI, *p* value**
Elective	258	88	134	64	0.73 (0.66, 0.82), *p* < 0.001
Emergent	8	3	6	3	1.06 (0.37, 3.00), *p* = 0.918
Urgent	16	5	68	33	5.99 (3.58, 10.02), *p* < 0.001
**Transposition of the great arteries**	**Count**	**(%)**	**Count**	**(%)**	**RR & 95%CI, *p* value**
Number of operations	19	6	22	11	1.63 (0.91, 2.94), *p* = 0.100
	**Median**	**IQR**	**Median**	**IQR**	***p* value**
Age at surgery, days	46	(11–62.5)	15	(11.2–25.5)	*p* = 0.047

na = not applicable. Associations highlighted in green are statistically significant.

Surgical risk scores, complication rates and mortality rates were available for the IQIC database and thus CHD procedures only (Table 3). RACHS-1 severity scores ranged from level 1–4 across both periods with no level 5 or 6 procedures performed. RACHS-1 levels 1–4 accounted for 13, 47, 34 and 6% of procedures in the pre-COVID-19 period and 15, 46, 32 and 8% of procedures in the peri-COVID-19 period, respectively. There was no significant change in RACHS-1 level proportions between periods. Delayed Sternal Closure (DSC) rates however were significantly higher in the peri-COVID-19 period (PR: 2.55, 95%CI: 1.13–5.74, *p* = 0.020). Stratification by neonatal status indicated that this association was not a result of confounding due to the increased incidence of neonatal procedures in this period (non-neonatal DSC PR: 3.20, 95%CI: 1.09–9.33, *p* < 0.05 vs. neonatal DSC PR: 1.10 95%CI: 0.34–3.59, *p* = 1.000).

**Table 3:** Congenital heart disease surgery – complexity and outcomes.

Period	Pre-COVID-19		Peri-COVID-19		% Change
**CHD surgeries (total)**	252		154		39%, IRR 0.61, *p* < 0.001
Complexity (CHD only)	**Count**	**(%)**	**Count**	**(%)**	**RR & 95%CI, *p* value**
RACHS-1 – Level 1	32	13	22	15	1.10 (0.66, 1.82), *p* = 0.716
RACHS-1 – Level 2	116	47	69	46	0.95 (0.76, 1.18), *p* = 0.647
RACHS-1 – Level 3	84	34	48	32	0.91 (0.68, 1.22), *p* = 0.538
RACHS-1 – Level 4	14	6	12	8	1.37 (0.65, 2.88), *p* = 0.407
Delayed sternal closure (DSC)	9	4	14	9	2.55 (1.13, 5.74), *p* = 0.020
Neonatal DSC	4/22	18	5/25	20	1.10 (0.34, 3.59), *p* = 1.000
Non-neonatal DSC	5/229	2.2	9/129	7.0	3.20 (1.09, 9.33), *p* = 0.025
**Complications**	**Count**	**(%)**	**Count**	**(%)**	**95% CI, *p* value**
Suspected bacterial sepsis	116	46	86	56	1.21 (1.00, 1.47), *p* = 0.055
Confirmed bacterial sepsis^b^	35	14	17	11	0.79 (0.46, 1.37), *p* = 0.404
Surgical site infection	19	8	8	5	0.69 (0.31, 1.54), *p* = 0.358
Cardiopulmonary bypass event	1	0.4	1	0.6	1.64 (0.10, 25.97), *p* = 1.000
Other	56	22	51	33	1.49 (1.08, 2.06), *p* = 0.016
Combined^a^	129	51	95	62	1.21 (1.01, 1.43), *p* = 0.039
**Mortality**	**Count**	**(%)**	**Count**	**(%)**	**95% CI, *p* value**
In hospital death	13	5	3	2	0.38 (0.11, 1.30), *p* = 0.107
RACHS-1 – Level 1	1/31	3.1	0/24	0.0	na
RACHS-1 – Level 2	7/110	6.0	1/68	1.4	0.24 (0.03, 1.93), *p* = 0.272
RACHS-1 – Level 3	3/81	3.6	1/47	2.1	0.58 (0.06, 5.45), *p* = 1.0
RACHS-1 – Level 4	2/12	14.3	1/11	8.3	0.58 (0.06, 5.66), *p* = 1.0
30-day mortality	15	6	7	5	2.18 (0.49, 9.62), *p* = 0.507
RACHS-1 – Level 1	1/31	3.1	0/23	0.0	na
RACHS-1 – Level 2	8/108	6.9	2/67	2.9	0.42 (0.09, 1.92), *p* = 0.408
RACHS-1 – Level 3	3/81	3.6	4/41	8.9	2.49 (0.58, 10.64), *p* = 0.388
RACHS-1 – Level 4	3/11	21.4	1/11	8.3	0.39 (0.05, 3.27), *p* = 0.706
Loss to follow up	5	2	9	6	2.95 (1.01, 8.63), *p* = 0.039

a Multiple complication categories permitted per procedure, but only 1 combined complication per procedure.

b Confirmed bacteremia on sterile blood culture.

na = not applicable. Associations highlighted in green are statistically significant.

The combined surgical complication rate was significantly higher in the peri-COVID-19 period, with 62% of procedures having complications compared with 51% in the pre-COVID-19 period (PR: 1.21, 95%CI: 1.01–1.43, *p* < 0.05). For complication categories multiple complications were possible per procedure; bacterial sepsis rates were high for both periods, 46% (116/252) in the pre-COVID-19 period and 56% (86/154) in the peri-COVID-19 period, however rates were similar between periods (PR: 1.21, 95%CI: 1.00–1.47, *p* = 0.055). Similarly, surgical site infection rates were similar between periods 8% (19/252) pre-COVID-19 and 5% (8/154) peri-COVID-19 (PR: 0.69, 95% CI: 0.31–1.54, *p* = 0.358), as where Cardiopulmonary bypass events, 0.4% (1/252) pre-COVID-19 and 0.6% (1/154) peri-COVID-19 (PR: 1.64, 95% CI: 0.10–25.97, *p* = 1.0). There was, however, a significant increase in “other complications” a heterogenous group of complications not related to bacterial sepsis, surgical site infection or cardiopulmonary bypass events. The other complication rate increased from 22% (56/252) in the pre-COVID-19 period to 33% (51/154) in the peri-COVID-19 period (PR: 1.49 95%CI: 1.08–2.06, *p* < 0.05).

There was no significant difference in in-hospital mortality, PR 0.38 (95% CI: 0.11–1.30, *p* = 0.107) or 30-day mortality, PR 2.18 (95% CI: 0.50–9.61, *p* = 0.506), however, loss-to-follow up rates at 6-weeks post-surgery were significantly higher in the peri-COVID-19 period than in the pre-COVID-19 period (PR: 2.95, 95%CI: 1.01–8.63, *p* = 0.039).

Unfortunately, the catheterisation laboratory was closed for renovations for 3 months in the pre-COVID-19 period and 5 months in the peri-COVID-19 period, as such, the following results are likely exaggerated. Cardiac catheterisations decreased by 42% (175 to 102) with no significant change in median age (3.3 years, IQR:0.9–7.6 pre-COVID-19 vs. 4.4 years, IQR:1.2–9 peri-COVID-19, *p* = 0.17), age category, or distribution of diagnoses. Similarly, there was no significant difference in the proportion of interventional procedures with 90 (51%) interventional cardiac catheterisations performed in the pre-COVID-19 period vs. 55 (54%) in peri-COVID-19 period (PR: 0.95, 95%CI: 0.76–1.20, *p* = 0.689). The complication rate was also similar between periods (12%, 21/175, pre-COVID-19 and 16%, 16/102, peri-COVID-19, PR: 1.31, 95%CI: 07.2–2.39, *p* = 0.384).

## 4. Discussion

Absolute clinic presentation, ward admission, and surgical and cardiac catheterisation procedure rates were all significantly reduced in the peri-COVID-19 period, when compared with the pre-COVID-19 period.

The 29% reduction (IRR 0.71, *p* < 0.001) in cardiac surgery is especially concerning when one considers that the South African pediatric cardiology and cardiothoracic surgery service are in general insufficient to meet the needs of the population. A 2006 audit of South African pediatric cardiac services demonstrated an overall pediatric cardiologist to population ratio of 1:4.5million people, well below the suggested ideal ratio of 1:500,000 (16), and that cardiothoracic surgical capacity was insufficient, able to meet the needs of <40% of children with CHD (8). Accordingly the COVID-19-related backlog cannot be cleared by returning to pre-COVID-19 capacity, additional capacity must be leveraged and evidence based strategies implemented to minimize complications and deaths in patients awaiting treatment.

For example, a recent United Kingdom study (19) of COVID-19 related backlog in adult-onset severe aortic stenosis patients showed that COVID-19 related restrictions had resulted in a significant backlog of patients. In this case service capacity was based on the pre-COVID-19 aortic stenosis incidence rate, thus it would not be possible to clear this backlog by returning to pre-COVID-19 capacity, instead extra capacity would need to be introduced. Mathematical modelling showed that a 20% increase in capacity would require 535 (434–666) days to clear the backlog with an associated mortality of 1,172 (466–1859) during this period. The model showed that if this capacity increase was supplemented by converting 40% of surgical cases to transcatheter aortic valve implantation, the backlog could be cleared within a year or 343 (281–410) days with fewer (784 [292–1,324]) deaths whilst awaiting treatment. This data-driven approach enabled researchers to optimize increases in capacity and minimize patient morbidity and mortality.

The 325% increase in urgent cases during the peri-COVID-19 period, 68 (33%) versus 16 (5%) in the pre-COVID-19 period, (PR: 5.99, 95%CI: 3.58–10.02, *p* < 0.001) indicates under-capacity of the Western Cape pediatric cardiology and cardiothoracic surgery service. By curtailing elective but necessary procedures, COVID-19 restrictions artificially lowered demand for cardiothoracic services, increasing the capacity of the system to deal with urgent cases. Thus, the higher absolute number of urgent cases treated in the peri-COVID-19 period better represents the true requirements of the Western Cape population. In fact, one could argue that the unique circumstances of the COVID-19 pandemic enabled the cardiac service to better treat patients at the point of physiological need, albeit at the expense of patients requiring elective procedures. This premise is corroborated by the sub-analysis of TGA-surgery. As expected, the absolute number of TGA-surgeries was similar between periods, however median age at surgery was significantly lower in the peri-COVID-19 period, 15 (11.2–25.5) days versus 46 (11–62.5) days (*p* < 0.05). In TGA early intervention is associated with improved outcomes (22) and many clinicians recommend that surgery be performed within 2–4 days of delivery (23). Unburdened by elective procedures the Western Cape paediatric cardiac service was able to intervene more timeously and significantly closer to the point of physiological need.

These data support previous studies which indicate the Western Cape cardiac service is running below capacity and as such the 29% reduction in surgical cases over the peri-COVID-19 period cannot be resolved by returning to pre-COVID-19 capacity. These data highlight the need for a detailed analysis of the composition of the Western Cape surgical backlog, surgical waiting times, and backlog morbidity and mortality rates. Additionally, they emphasize the need for the development of a fully informed and mathematically modelled strategy to increase capacity and reduce backlog whilst ensuring minimal rates of morbidity and mortality.

Direct measures of surgical complexity showed no significant change between periods, with no significant change in the proportion of procedure-type or RACHS-1 risk categories performed between periods. RACHS-1 risk categories 2 and 3 account for 81 and 78% of all procedures for the pre-COVID-19 and peri-COVID-19 periods, respectively. The predominance of risk category 2 and 3 procedures may mask subtle changes in surgical complexity between periods. Additionally, some indirect measures of surgical complexity showed significant differences.

Median age at surgery was significantly lower, 7.2 months (2.04–20.4) peri-COVID-19 versus 10.8 months (4.8–49.2) pre-COVID-19 (*p* = 0.001), related to a significant increase in neonatal procedures (PR: 2.48, 95% CI: 1.48–3.99, *p* < 0.001) and a decrease in patients aged 5 to 12 (PR: 0.57, 95% CI: 0.34–0.88, *p* < 0.05). Neonatal cardiac surgery is technically challenging and lesions requiring neonatal intervention are typically more severe (24). Despite this, internationally there is an increase in the number of neonatal procedures (25) Similarly, over the three year period prior to the COVID-19 pandemic, there was a non-significant increase in neonatal procedures in the Western Cape pediatric cardiothoracic service (PR: 1.17, 95% CI: 0.70–1.96, *p* = 0.5) (26). A major limitation of uncontrolled pre-post studies is that the exposure-group is not compared with a contemporaneous control-group, as such associations may be confounded by non-contemporaneous control bias. It is possible that this general increase in neonatal procedures is confounding this association, however, we believe that this trend alone could not be responsible for the more than two-fold increase in the proportion of neonatal procedures over the period. Instead, we believe this is another indicator of the prioritization of urgent neonatal cases in a system typically operating below capacity to meet the needs of the population. During the peri-COVID-19 period artificially lowered numbers of elective procedures increased capacity for urgent neonatal procedures on patients that otherwise may have died or would have been operated on in infancy but at higher risk.

Delayed sternal closure (DSC) may be indicated after pediatric cardiac surgery, typically in the setting of severely impaired cardiac function, is often used after neonatal cardiac procedures (27) and is a good indicator of surgical complexity. Indications and applications of the technique vary from center to center (27) in the Western Cape pediatric cardiothoracic service the decision to delay sternal closure is handled on a case by case basis, however neonates accounted for 42% of all cases of DSC reported over the study period. The proportion of cases requiring DSC in the peri-COVID-19 period was more than double that reported in the pre-COVID-19 period (PR: 2.55, 95%CI: 1.13–5.74, *p* = 0.020), indicating an increased proportion of severe cases in this period. In addition, when controlled for neonatal status through stratification the strength of this association increased (PR: 3.20, 95%CI: 1.09–9.33, *p* < 0.05) indicating that this finding was not a result of the increased proportion of neonatal procedures in the peri-COVID-19 period and is a result of the increased proportion of urgent and complicated cases.

There was no significant change in the number or length of ICU admissions, however, total length of stay (combined ward and ICU) was significantly longer in the peri-COVID-19 period, 6 (IQR: 2–14) days versus 3 (IQR: 1–9) days in the pre-COVID-19 period (*p* < 0.001). Complication rates were higher in the peri-COVID-19 period (PR: 1.21, 95%CI: 1.01–1.43, *p* < 0.05). Bacterial sepsis rates were high for both periods with a non-significant increase in the peri-COVID-19 period. The definition of bacterial sepsis, however, was broad and based on the IQIC definition of: “presumed or confirmed bacterial sepsis with fever or hypothermia, tachycardia, hypotension, tachypnoea, leukocytosis or leukopenia.” Positive blood cultures were not a requirement and bacterial sepsis, secondary to other infections such as pneumonia, catheter associated bloodstream infections, or surgical site infections were also included. Culture-confirmed bacterial sepsis rates were lower overall with a non-significant decrease in the peri-COVID-19 period. Surgical site infection rates and cardio-pulmonary bypass event rates were similar between periods. There was, however, a significant increase in complications not related to bacterial sepsis, surgical site infection or cardiopulmonary bypass events (PR: 1.49 95%CI: 1.08–2.06, *p* < 0.05) in the peri-COVID-19 period, this association was not directly related to an increase in viral illness or COVID-19 cases during the peri-COVID-19 period, however, may have been a result of pandemic related stressors or increased complexity of cases in this period.

Interestingly, despite the increase in the complexity and the proportion of urgent cases, both in-hospital and 30-day mortality rates remained stable across periods and compare favorably with international benchmarks (20). It is possible that systemic changes in peri-operative care implemented during the peri-COVID-19 period were protective. For example, non-essential procedures and visits were cancelled and personnel who worked in these areas redeployed to critical areas (7). This combined with reduced-surgical caseloads likely resulted in an improved ratio of health care practitioners to patients in critical care areas. Additionally, stricter bedside hygiene practices including the use of personal protective equipment, rigorous hand washing protocols and isolation precautions were likely protective. Moreover, paediatric admissions accounted for only 2.9% of all South African COVID-19 admissions (28) thus these changes may have been in excess of actual requirements. Also, it is possible that these findings indicate the benefits of earlier surgical intervention.

COVID-19 restrictions have had negative impacts on both undergraduate and post-graduate medical training. Effects on post-graduate training were primarily related to reduced caseload and exposure, diversion of staff to COVID-19 related activities, cancelled academic conferences and professional fora, and suspension of international training (29). For example, anesthetic trainees across 6 continents, reported that reduced caseload, sub-specialty experience, and supervised procedures impaired learning (30). Additionally, in the United States, reduced patient volumes resulted in reduced procedural learning opportunities, leading some residency programs to waive minimum procedure requirements for graduating residents (29). The 30% reduction in surgical procedures and 40% reduction in cardiology admissions during the peri-COVID-19 period clearly indicate reduced caseloads for Western Cape pediatric cardiology and cardiothoracic surgery residents. Additionally, despite RACHS-1 scores being similar for both periods, the higher proportion of urgent cases, neonatal procedures, and increased admission-duration in the peri-COVID-19 period indicates an increase in severe or complicated cases. These cases would likely necessitate increased senior responsibility, further impacting cardiothoracic surgery training. Accordingly, the higher proportion of complex and technically challenging cases together with the significant reduction in caseload may have implications on cardiothoracic surgical training and both qualitative and quantitative studies of cardiothoracic surgical training during the peri-COVID-19 period should be considered.

## Data availability statement

The data analyzed in this study is subject to the following licenses/restrictions: Data are available upon reasonable request from TA. Requests to access these datasets should be directed to thomas.aldersley@uct.ac.za.

## Ethics statement

The studies involving human participants were reviewed and approved by Human Research Ethics Committee, University of Cape Town. Written informed consent from the participants’ legal guardian/next of kin was not required to participate in this study in accordance with the national legislation and the institutional requirements.

## Author contributions

LZ, JL, and TA: conceptualization. LZ, JL, AB, and LS: supervision. TA, PH, and SS: data curation. TA: formal analysis and writing – original draft. TA, AB, PH, JL, GC, RD, BF, RM, HP, SS, LS, and LZ: writing – critical review and editing. LZ: funding acquisition. All authors contributed to the article and approved the submitted version.

## Funding

LZ is funded by the South African Medical Research Council (SAMRC) through its Division of Research Capacity Development under the Mid-Career Scientist Programme from funding received from the South African National Treasury. The content hereof is the sole responsibility of the authors and do not necessarily represent the official views of the SAMRC. LZ also receives support from the National Research Foundation of South Africa (NRFSA), as well as the UK Medical Research Council (MRC) and the UK Department for International Development (DFID) under the MRC/DFID Concordat agreement, via the African Research Leader Award (MR/S005242/1).

## Conflict of interest

The authors declare that the research was conducted in the absence of any commercial or financial relationships that could be construed as a potential conflict of interest.

## Publisher’s note

All claims expressed in this article are solely those of the authors and do not necessarily represent those of their affiliated organizations, or those of the publisher, the editors and the reviewers. Any product that may be evaluated in this article, or claim that may be made by its manufacturer, is not guaranteed or endorsed by the publisher.

## Abbreviations

CHD: Congenital heart disease
DSC: Delayed sternal closure
IQIC: International Quality Improvement Collaborative for Congenital Heart Disease
OPD: Out-patient department
Peri-COVID-19: The research period during the COVID-19 pandemic (01/03/2020–28/02/2021)
Pre-COVID-19: The research period prior to the COVID-19 pandemic (01/03/2019–29/02/2020)
RACHS-1: Risk Adjustment for Congenital Heart Surgery 1 score
95%CI: 95% confidence interval
IQR: Interquartile range
IRR: Incidence rate ratio
PR: Prevalence ratio

## References

[1] Department of Co-Operative Governance and Traditional Affairs (South Africa). Disaster management act no. 57 of 2002. Regulations issued in terms of section 27(2) of the disaster management act, 2002. Government gazette no. 43107 2020 (published under government notice 318). March 18, 2020.

[2] Department of Transport (South Africa). National Ports act, 2005 (act no. 12 of 2005) regulations in terms of sections 80 (1) (G) 2020. Government gazette no. 43103 2020 (published under government notice 173). March 18, 2020.

[3] Department of Transport (South Africa). International air services act, 1993 (act no. 60 of 1993) regulations, in terms of section 43 (1) (h) 2020. Government gazette no. 43105 2020 (published under government notice 173). March 25, 2020.

[4] Department of Co-operative Governance and Traditional Affairs (South Africa). Disaster management act no. 57 of 2002. Regulations issued in terms of section 27(2) of the disaster management act, 2002. Government gazette no. 43148 2020 (published under government notice 398). March 25, 2020.

[5] Department of Trade and Industry (South Africa). Competition act no. 89 of 1998. Covid-19 block exemption for the healthcare sector, 2020. Government gazette no. 43114 2020 (published under government notice 349). March 19, 2020.

[6] Chu K Smith M Steyn E Goldberg P Bougard H Buccimazza I. Changes in surgical practice in 85 south African hospitals during COVID-19 hard lockdown. S Afr Med J. (2020) 110:916–9. doi: 10.7196/SAMJ.2020.v110i9.15014, PMID: 32880278

[7] Parbhoo A Numanoglu A Argent A Franken M Mukosi M McCulloch M. COVID-19: experience of a tertiary children’s hospital in Western Cape Province. South Africa S Afr Med J. (2021) 111:295–8. doi: 10.7196/SAMJ.2021.v111i4.15539, PMID: 33944758

[8] Hoosen E Cilliers A Hugo-Hamman C Lawrenson J Brown S Harrisberg J. Audit of paediatric cardiac services in South Africa. SA Heart. (2010) 7:4–9. doi: 10.24170/7-1-1962 21678733

[9] World Health Organization. The Impact of the COVID-19 Pandemic on Noncommunicable Disease Resources and Services: Results of a Rapid Assessment. Geneva: WHO (2020).

[10] Chu KM Marco J Bougard H Strauss CP Bertels L Victor AE. Estimating the surgical backlog from the COVID-19 lockdown in South Africa: a retrospective analysis of six government hospitals. S Afr Med J. (2021) 111:685–8. doi: 10.7196/SAMJ.2021.v111i7.15686, PMID: 34382554

[11] Rubino AS De Santo LS Pisano A di Mauro M Benussi S Borghetti V. Cardiac surgery practice during the COVID-19 outbreak: a multicentre national survey. Eur J Cardiothorac Surg. (2021) 59:901–7. doi: 10.1093/ejcts/ezaa436, PMID: 33657222 PMC7989504

[12] Utria AF Javid PJ Chen J Rice-Townsend SE. Impact of COVID-19 on procedure volume at a tertiary pediatric hospital. Am J Surg. (2021) 221:1259–61. doi: 10.1016/j.amjsurg.2021.03.003, PMID: 33707079 PMC7933785

[13] Hoosen EG Cilliers AM Brown S Mitchell B. Improving access to pediatric cardiac Care in the Developing World: the South African perspective. Curr Treat Pediatrics. (2022) 8:1–10. doi: 10.1007/s40746-022-00247-w PMC9137262 37521172

[14] Brown S Pepeta L. Paediatric cardiology in South Africa 2016: quo vadis? SA Heart. (2016) 13:2–4. doi: 10.24170/13-1-1685

[15] Hoosen E Cilliers A Hugo-Hamman C Lawrenson J Brown S Harrisberg J. Optimal paediatric cardiac services in South Africa-what do we need? Statement of the Paediatric cardiac Society of South Africa: paediatric cardiac services. SA Heart. (2010) 7:10–6. doi: 10.24170/7-1-1963

[16] Hall R More R Camm J Swanton H Gray H Flint J. Fifth report on the provision of services for patients with heart disease. Heart. (2002) 88:3–56. doi: 10.1136/heart.88.suppl_3.iii1 12368237 PMC1876276

[17] Department of Statistics (South Africa). P0305, recorded live births, 2019. October 19, 2020.

[18] Jacobs JP Mavroudis C Quintessenza JA Chai PJ Pasquali SK Hill KD. Reoperations for Pediatric and Congenital Heart Disease: An Analysis Of The Society Of Thoracic Surgeons (STS) Congenital Heart Surgery Database. Seminars in Thoracic and Cardiovascular Surgery: Pediatric Cardiac Surgery Annual. Amsterdam: Elsevier (2014). 10.1053/j.pcsu.2014.01.006 PMC4276147 24725711

[19] Stickels CP Nadarajah R Gale CP Jiang H Sharkey KJ Gibbison B. Aortic stenosis post-COVID-19: a mathematical model on waiting lists and mortality. BMJ Open. (2022) 12:e059309. doi: 10.1136/bmjopen-2021-059309, PMID: 35710248 PMC9207579

[20] Jenkins KJ Gauvreau K Newburger JW Spray TL Moller JH Iezzoni LI. Consensus-based method for risk adjustment for surgery for congenital heart disease. J Thorac Cardiovasc Surg. (2002) 123:110–8. doi: 10.1067/mtc.2002.119064, PMID: 11782764

[21] R Core Team. R: A language and environment for statistical computing. 4.2.0 ed. Vienna, Austria: R Foundation for Statistical Computing (2022).

[22] Anderson BR Ciarleglio AJ Hayes DA Quaegebeur JM Vincent JA Bacha EA. Earlier arterial switch operation improves outcomes and reduces costs for neonates with transposition of the great arteries. J Am Coll Cardiol. (2014) 63:481–7. doi: 10.1016/j.jacc.2013.08.1645, PMID: 24184243

[23] Villafañe J Lantin-Hermoso MR Bhatt AB Tweddell JS Geva T Nathan M. D-transposition of the great arteries: the current era of the arterial switch operation. J Am Coll Cardiol. (2014) 64:498–511. doi: 10.1016/j.jacc.2014.06.1150, PMID: 25082585 PMC4340094

[24] Krishnamurthy G Ratner V Bacha E. Neonatal cardiac care, a perspective. Seminars in thoracic and cardiovascular surgery: Pediatric cardiac surgery annual. Amsterdam: Elsevier (2013). 10.1053/j.pcsu.2013.01.007 23561814

[25] Hasegawa T Masuda M Okumura M Arai H Kobayashi J Saiki Y. Trends and outcomes in neonatal cardiac surgery for congenital heart disease in Japan from 1996 to 2010. Eur J Cardiothorac Surg. (2017) 51:301–7. doi: 10.1093/ejcts/ezw302, PMID: 28186248

[26] Du Toit D Lenise S Salie S Perkins S Basera W Aldersley T. Outcomes following Neonatal Cardiac Surgery in Cape Town, South Africa. (n.d). [Submitted for publication]. 10.1177/21501351241268559 39205439

[27] Özker E Saritaş B Vuran C Yörüker U Ulugöl H Türköz R. Delayed sternal closure after pediatric cardiac operations; single center experience: a retrospective study. J Cardiothorac Surg. (2012) 7:1–6. doi: 10.1186/1749-8090-7-102 23031425 PMC3514162

[28] Kufa T Jassat W Cohen C Tempia S Masha M Wolter N. Epidemiology of SARS-CoV-2 infection and SARS-CoV-2 positive hospital admissions among children in South Africa. Influenza Other Respir Viruses. (2022) 16:34–47. doi: 10.1111/irv.12916, PMID: 34796674 PMC9664941

[29] Edigin E Eseaton PO Shaka H Ojemolon PE Asemota IR Akuna E. Impact of COVID-19 pandemic on medical postgraduate training in the United States. Med Educ. (2020) 25. doi: 10.1080/10872981.2020.1774318 PMC7448893 32493181

[30] Sneyd JR Mathoulin SE O'Sullivan EP So VC Roberts FR Paul AA. Impact of the COVID-19 pandemic on anaesthesia trainees and their training. Br J Anaesth. (2020) 125:450–5. doi: 10.1016/j.bja.2020.07.011, PMID: 32773215 PMC7377727

